# Effect of Turning Amount on Metallurgical Qualities and Mechanical Properties of GH4169 Superalloy

**DOI:** 10.3390/ma12111852

**Published:** 2019-06-07

**Authors:** Jinglong Qu, Shufeng Yang, Zhengyang Chen, Jinhui Du, Jingshe Li, Di Wang

**Affiliations:** 1 Beijing Central Iron and Steel Research Institute, Beijing 100081, China; QJL13810256459@163.com (J.Q.); superalloy_1@163.com (J.D.); 2 School of Metallurgical and Ecological Engineering, University of Science and Technology Beijing, Beijing 100083, China; chenzhengyang@xs.ustb.edu.cn (Z.C.); g20188273@163.com (D.W.); 3 Beijing Key Laboratory of Special Melting and Preparation of High-End Metal Materials, Beijing 100083, China

**Keywords:** GH4169 superalloy, vacuum arc remelting, turning amount, inclusions, mechanical properties

## Abstract

The determination of an appropriate amount of turning for superalloy ingot surfaces, in a scientific and reasonable manner, is vital to the improvement of the metallurgical quality and comprehensive performance of superalloy ingots. In the present study, scanning electron microscopy with energy-dispersive spectroscopy, a high-temperature testing machine, a Brinell hardness tester and the Image-Pro Plus software were used to analyze and compare the types and amounts of inclusions, the average area of the (Al,Mg)O inclusions, and the mechanical properties of points at different distances from the edge of the GH4169 superalloy vacuum arc remelting (VAR) ingot edge. The effects of the amount of turning to which the superalloy is subjected, the metallurgical qualities, and the mechanical properties were systematically studied. The results showed that the five inclusion types did not change as the sampling locations moved away from the ingot edge, but the amount of inclusions and the average area of the (Al,Mg)O inclusions first decreased and then stabilized. Similarly, the tensile strength, elongation, section shrinkage, hardness, and fatigue life first increased and then stabilized. Finally, this experiment tentatively determined that an appropriate amount of turning for a GH4169 superalloy ingot is 36–48 mm.

## 1. Introduction

GH4169 superalloy exhibits excellent resistance to oxidation, corrosion, and fatigue, while exhibiting good plasticity and a tensile strength in the range of ~253–650 °C [1,2,3]. It is ideal for the manufacture of a variety of complex parts. In the fields of aerospace and aviation engineering, it has been widely applied to the manufacturing of key parts such as turbine disks and blades [4,5,6].

As aircraft engines have become safer, more economical, and lighter, it has gradually been determined that differences in the surface qualities of superalloy parts will seriously affect their overall mechanical properties. This poses a significant threat to the safety and stability of the alloy while in service [7,8,9]. Some researchers have improved the surface quality of superalloys by adjusting the processing parameters, the processing environment, and the tools used for machining. For example, Suárez et al. [10] studied the effects of ultrasonic-assisted face milling on the surface integrity and fatigue life of Inconel 718 superalloy. They found that, during machining, the ultrasonic-assisted milling can effectively improve the surface quality of the alloy and increase its fatigue life by 14.74%. Pusavec et al. [11] compared and analyzed the influence of low-temperature processing and traditional dry processing on the surface quality of Inconel 718 high-temperature alloy, and found that low-temperature processing could effectively improve the surface roughness of the alloy and reduce the thickness of its process-hardened layer. Feyzi et al. [12] applied a new mixed-processing technology to the surface treatment of Inconel 718 superalloy and found that the cutting force required for the mixed processing was only 7–14% of that required for conventional processing, while the surface quality of the alloy processed using the mixed processing method was 88–93% better.

In addition, some researchers have developed theoretical models for the grinding process to provide a theoretical basis for controlling and improving the surface quality of the alloy. For example, Hechker et al. [13] analyzed the influence of the grinding depth, rate ratio, equivalent diameter of the workpiece, and the microstructure of the grinding wheel on the grinding surface roughness, and established a model for predicting the grinding surface roughness, based on relevant experimental data. Suárez et al. [14] theoretically analyzed the temperature changes, wear changes, shear stress changes and other aspects of the turning inserts in the surface turning technology of Inconel 718 superalloy based on different cooling-water pressures, established a mathematical model of the temperature distribution in the turning inserts, and completed the related model verification. Guo et al. [15], using the traditional grinding model for an alumina wheel and experimental results obtained for the influence of the grinding wheel wear and processing parameters on superalloy grinding, established a grinding power model for a cubic boron nitride (CBN) wheel acting on a superalloy. D’Addona et al. [16] combined the heat transfer process for the high-pressure-coolant grinding of an Inconel 718 superalloy surface with numerical simulation software (Ansys) to establish a mathematical model of tool temperature distribution in a grinding process with high-pressure cooling. The influence of the coolant pressure on the tool temperature and the relationship between the tool and friction coefficient of the alloy surface were analyzed. 

To summarize, researchers have improved the alloy surface quality by promoting theoretical research into the grinding process and optimizing the processing technology. However, there have been very few reports on the impact of the amount of turning applied to Inconel 718 superalloy on its performance. Steel mills rely on operational experience or an ingot’s surface finish as the standard for measuring the turning quality of a vacuum arc remelting (VAR) ingot, ignoring the slag discharge effect of the VAR process [17,18,19]. Even if the surface finish of the ingot satisfies the technological requirements, a large number of types and amounts of inclusions remain on the surface, which seriously limit the further improvement of the alloy’s performance [20,21]. To avoid this phenomenon, it is particularly important to study the influence of different amounts of turning on the metallurgical qualities of a GH4169 superalloy ingot. In the present study, a triple-melting technique was used to refine the GH4169 superalloy. Then, the mechanical properties of samples taken from points at different distances from the edge of the VAR ingot were tested, the average area changes of the inclusions type and amounts, as well as the (Al,Mg)O inclusions, were observed and counted. The effects of the location from which the samples were taken on the inclusion distribution and mechanical properties were analyzed and compared. This provided a theoretical engineering guidance and values for an appropriate turning amount for a GH4169 superalloy ingot.

## 2. Materials and Methods

### 2.1. Melting

The GH4169 superalloy was melted by vacuum induction melting (VIM). The main furnace charge proportion of GH4169 superalloy is listed in Table 1, which conforms to the AMS specifications. Once the alloy had completely melted, the solution temperature was 1460 ± 10 °C and the refining time was 60 min. The vacuum during the melting and refining was kept below 1.5 Pa. After refining, the composition and temperature of the liquid alloy in the crucible were adjusted, and trace alloy elements were added. Then, an alloy ingot with a diameter of 360 mm was cast at 1410 ± 10 °C. 

The Ø360 mm ingot surface was polished to reduce the effects of the refractory and surface-oxide coatings on the purity of the ingot produced by the electro-slag remelting smelting process. Then, the ingot of an GH4169 superalloy was re-melted by electroslag remelting (ESR) with CaF_2_, Al_2_O_3_, and CaO as the slag components. During the ESR process, the argon (protective gas) was injected at a flow rate of 60 ± 2 L/min. The operation mode involving the addition of slag while melting slag was adopted in the early stages of smelting, with slag-melting of the ingot requiring about 60 min. The melt rate of the refining process was steady at a rate of 6.2 ± 0.5 kg/min. Ultimately, a Ø440 mm alloy ingot was obtained.

**Figure 1:**
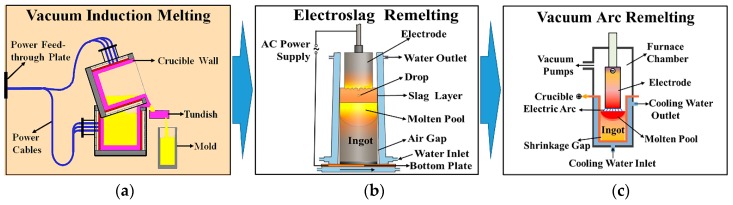
Melting technology. (**a**) Vacuum induction melting; (**b**) Electroslag remelting; (**c**) Vacuum arc remelting.

The ESR-refined ingot was turned to avoid secondary contamination by the slag. The ESR ingot was re-melted by VAR, where the vacuum of VAR process was less than 0.5 Pa. The melt rate was maintained at 6.5–7.5 kg/min by controlling the voltage and current to form the molten pool, with the total duration being about 55 min. The melt rate was steady and gradually decreased at a constant rate of 4.1–5.3 kg/min during the steady melting stage. A Ø508 mm alloy ingot was obtained as a result. The triple smelting technology is shown in Figure 1.

### 2.2. Experiment

**Figure 2:**
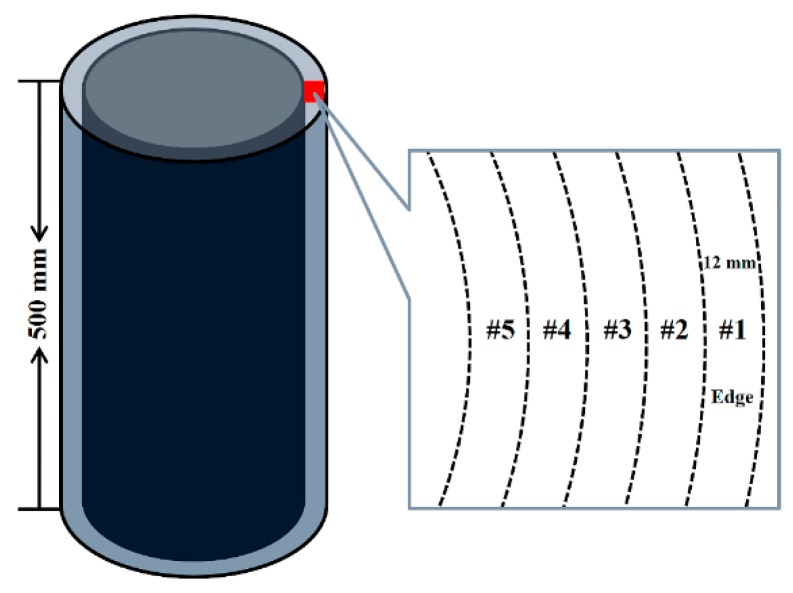
Sampling area.

A 500 mm alloy bar was taken from the top of the ingot casting, after which samples were taken from the edge of the bar (every 12 mm around the edge, giving a total of five regions named, respectively, #1, #2, #3, #4, and #5). Six samples measuring Ø12 mm × 66 mm and three measuring Ø12 mm × 110 mm (tensile samples), and another three measuring Ø12 mm × 15 mm (metallographic specimens) were taken (Figure 2). Subsequently, the mechanical properties of the five groups of tensile samples and metallographic specimens taken from each of the regions were tested by the cupping machine (tensile strength tests, elongation tests, and section shrinkage tests were carried out at 25 °C and at 650 °C), the Brinell hardness tester (hardness tests were carried out at room temperature) and the fatigue-testing machine (fatigue life tests were carried out at 455 °C).

Fifteen metal samples (taken from five different regions, with three samples being taken from each region) were mechanically polished. The inclusions were characterized by scanning electron microscopy (SEM) equipped with energy dispersive spectroscopy (EDS) module Phenom ProX (Phenom World, Eindhoven, The Netherlands). Each sample was selected, on the basis of 100 metallographic photographs to obtain accurate inclusion data. The Image-Pro Plus 6.0 software (Media Cybernetics, Inc., Rockville, MD, USA) was used to analyze the distribution of the inclusions in five groups of samples, as well as the average area of the (Al,Mg)O inclusions.

## 3. Results and Discussion

### 3.1. Analysis of Inclusion Formation

**Figure 3:**
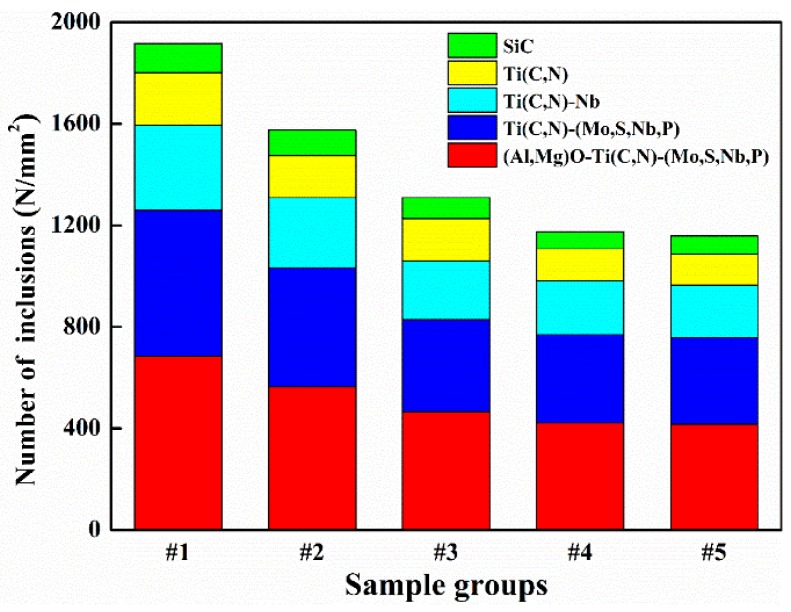
Numbers of different types of inclusions per unit area.

**Figure 4:**
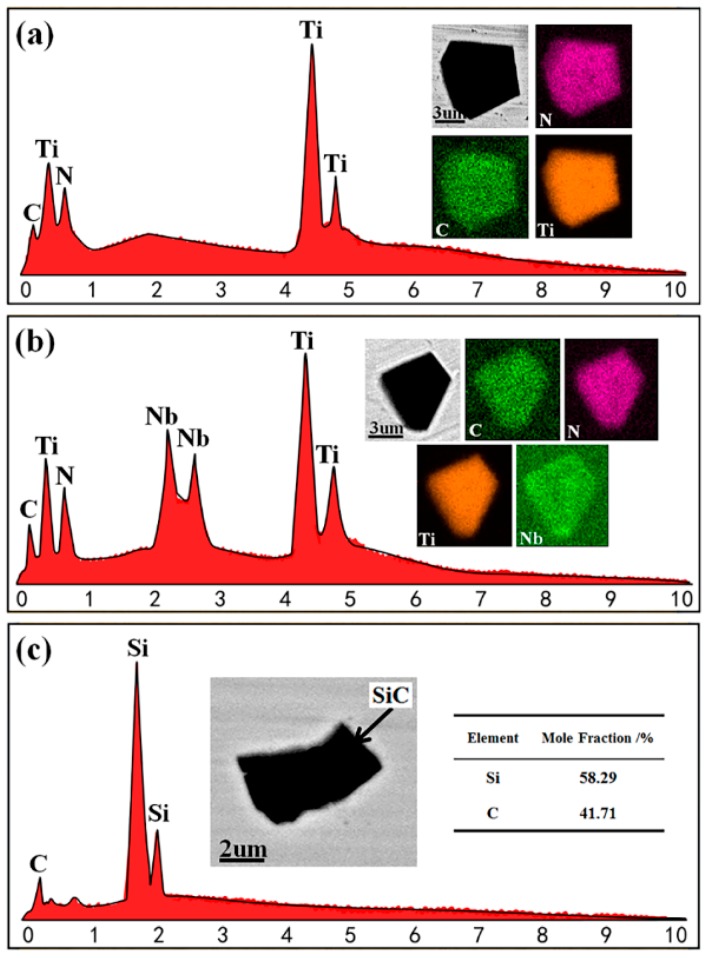
SEM images and energy dispersive spectroscopy (EDS) mapping of inclusions. (**a**) Ti(C,N); (**b**) Ti(C,N)-Nb; (**c**) SiC.

**Figure 5:**
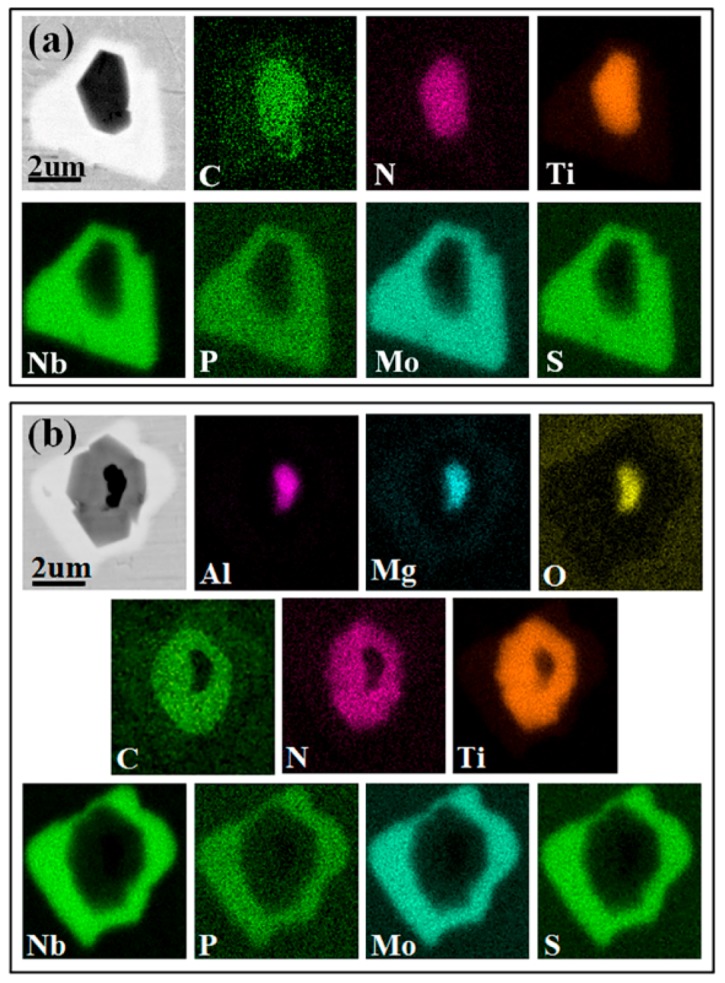
SEM images and EDS mapping of multi-layer inclusions. (**a**) Ti(C,N)-(Nb,P,Mo,S); (**b**) (Al,Mg)O-Ti(C,N)-(Nb,P,Mo,S).

The relevant inclusion results (types and amounts) are shown in Figure 3, Figure 4 and Figure 5. As shown in Figure 3, five types of inclusion were identified in all five samples: Ti(C,N) composite inclusions, Ti(C,N)-Nb composite inclusions, SiC inclusions, Ti(C,N)-(Nb,P,Mo,S) composite inclusions, and (Al,Mg)O-Ti(C,N)-(Nb,P,Mo,S) composite inclusions. However, the distribution of the number of inclusions per unit area in the five groups of samples was quite different; for example, samples #1 to #3 showed an obvious decreasing trend, with the value decreasing from 1917 to 1310 N/mm^2^. Compared with the #3 sample, the distribution of the number of inclusions in samples #4 and #5 decreased further, but the degree of decrease was smaller, with both fluctuating within a range of 1166 ± 10 N/mm^2^. Obviously, the distribution of the number of inclusions gradually decreases and tends to stabilize as the distance between the sampling location and the ingot edge increases. Therefore, it can be tentatively determined that, for a GH4169 superalloy ingot, a turning amount of 36–48 mm is appropriate. 

As shown in Figure 4a, the Ti in the alloy liquid combines with N and C to form TiN and TiC. The chemical reactions for TiN and TiC are shown in Equations (1) and (2). The TiN had a lower standard Gibbs free energy of formation than the TiC when the temperature of the molten steel was below 1450 °C; therefore, it preferentially nucleated. By acting as a nucleation core, the fine TiN reduces the undercooling required for TiC precipitation, leading to the aggregation and growth of TiC inclusions on the TiN surface [22]. In addition, both TiN and TiC have a NaCl-type structure with a small difference in the ambiguity constants (a_TiN_ = 0.4241 nm, a_TiC_ = 0.4329 nm). During the process of solidification, the TiN and TiC tend to form TiC–TiN composite inclusions without exhibiting distinct nucleation centers [23].

(1) Ti(l)+[N]=TiN(s)

(2) Ti(l)+C(s)=TiC(s)

Figure 4b shows that these types of inclusions have more Nb elements than Ti(C,N) composite inclusions. Based on an analysis of Ti(C,N) composite inclusions and the fact that the GH4169 superalloy contains elemental Nb, the Nb partially or totally replaces the Ti and C to form NbC [24]. With TiN as the nucleation core, it begins to accumulate and grow on its surface. In addition, the NbC, TiC and TiN possess surface-centered cubic structures and have the same structural gap phase, making them easy to form Ti(C,N)-Nb composite inclusions [24,25]. Consequently, the Ti(C,N)-Nb composite inclusions do not exhibit distinct nucleation centers.

Figure 4c shows that, during the VIM of the GH4169 superalloy, carbon monoxide forms from C and O in the alloy solution, thus removing the oxygen, nitrogen, and hydrogen from the solution. In the liquid metal, the relatively poor-quality CO gas decreases as the oxygen content gradually decreases. Thus, some CO is attached to the inside of the crucible wall containing elemental Si, causing the CO gas to react with Si to from SiC inclusions [26]. In addition, the graphite used in the ingot mold to facilitate VIM ingot demolding may react with the Silicon in the GH4169 superalloy to form SiC inclusions [27].

Figure 5a,b shows that the outermost layer of the two kinds of composite inclusions is uniformly surrounded by elemental Nb, P, Mo, and S. The main difference is that one kind of composite inclusion takes the (Al,Mg)O inclusions as the nucleation core, while the other kind takes the Ti(C,N) inclusions as the nucleation core. This is caused by the VIM smelting process adding elementary elements such as C and Al to the alloy liquor, leading to the erosion of the crucible wall which increases with the smelting time. The crucible material is usually fabricated from magnesia, such that it is difficult to prevent MgO from migrating from the crucible into the alloy liquor, which then leads to a reaction between the MgO and Al to form (Al,Mg)O inclusions [25]. As a heterogeneous nucleation core, the generated (Al,Mg)O inclusions result in TiN and TiC precipitation occurring with less undercooling. Combining this with the results of an analysis of the Ti(C,N) composite inclusions in our previous study, it is clear that Ti(C,N) will precipitate and grow on the surface of the (Al,Mg)O inclusions. At this time, Ti(C,N) inclusions are also present in the metal solution. The (Al,Mg)O-Ti(C,N)-(Nb,P,Mo,S) composite inclusions and Ti(C,N)-(Nb,P,Mo,S) composite inclusions could be formed as a result of different types of inclusions colliding or combining, because of the circulation and convection of the molten metals [28].

### 3.2. Analysis of (Al,Mg)O Inclusions

**Figure 6:**
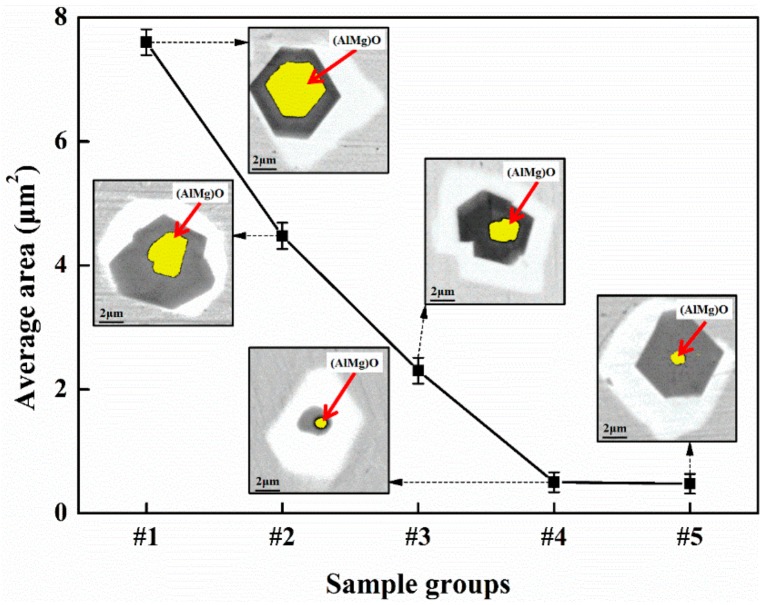
Average area of (Al,Mg)O inclusions in (Al,Mg)O-Ti(C,N)-(Nb,P,Mo,S) inclusions.

In the present study, the distribution of the inclusions in the five sample groups was analyzed. It was found that the average area of the (Al,Mg)O inclusions at the center varied greatly with the (Al,Mg)O-Ti(C,N)-(Nb,P,Mo,S) composite inclusions in the different regions, so a detailed analysis was undertaken. The average area of the (Al,Mg)O inclusions is shown in Figure 6. The average area a of (Al,Mg) O inclusions in sample #1 is the largest, at 7.61 µm^2^. Compared with sample #1, the average area a of the (Al,Mg)O inclusions in samples #2, #3, and #4 decreases significantly, with the value being only 0.59, 0.30, and 0.07 times that of sample #1. The average area of the (Al,Mg)O inclusions in samples #4 and #5 is in a range of 0.49 ± 0.02 µm^2^.

According to the above analysis, the average area a of the (Al,Mg)O composite inclusions first decreases and then stabilizes as the distance between the sampling area and the ingot edge increases, with the transition basically being similar to that of the number of inclusions in each of the regions. This is because, in the VAR smelting, the metal liquids in the molten pool are affected by gravity, buoyancy, and the Lorentz and Coulomb forces, such that there are different flow patterns in the molten pool, giving rise to the phenomenon of "slag discharge". That is, inclusions or impurities move outwards along the surface of the molten pool [29,30], thus causing a large number of inclusions to accumulate in the area around its edge. Moreover, the collision, aggregation, and growth of the (Al,Mg)O composite inclusions decreases as the distance between the sampling region and the edge of the ingot increases. Although some inclusions adhere to the side wall of the mold as a result of the "slag discharge" from the molten pool, some inclusions continue to be concentrated on the surface of the solidified ingot. This, combined with the VAR smelting process, results in the smelting characteristics of O, N, and H in the ingot being suppressed. That is, the content and quantity distribution of (Al,Mg)O complex inclusions can be reduced by reducing the O content in the ingot [18,28]. Thus, we can conclude that the number of inclusions per unit area and the average area of the (Al,Mg)O composite inclusions gradually decrease and tend to stabilize as the distance between the sampling location and the edge of the ingot increases. Therefore, the amount of turning for a GH4169 superalloy ingot can be tentatively determined to be 36–48 mm.

### 3.3. Analysis of Mechanical Properties

**Figure 7:**
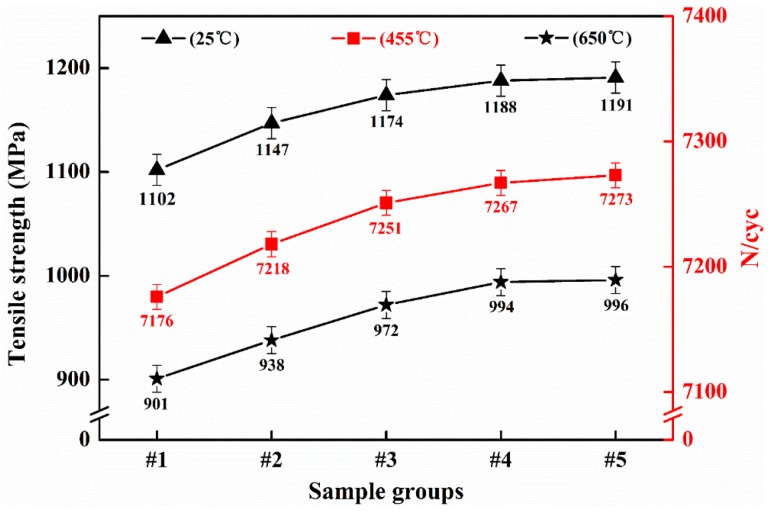
Tensile strength and fatigue life of superalloy sample groups.

Tensile tests, elongation tests, section shrinkage tests, hardness tests and fatigue life tests were carried out at both room temperature and high temperature on the five groups of samples taken from each of the regions. The results are shown in Table 2 and Figure 7. Table 2 shows that, as the sample group moves away from the ingot edge gradually, its elongation (at 25 °C and at 650 °C) increases from 13.0% to 15.5%, and from 24.0% to 29.0%, respectively; the section shrinkage (at 25 °C and 650 °C) increases from 28.0% to 32.0%, and from 55.0% to 60.0%, respectively. The hardness increases from 442 HB to 448 HB, but there is little difference between samples #4 and #5. Figure 7 shows that the average tensile strength of sample #1 is lowest when tensile deformation occurs at 25 °C, with a value of 1102 MPa. The average tensile strength of samples #2, #3, #4, and #5 increases by 45, 72, 86, and 89 MPa, respectively, relative to sample #1. The average tensile strengths of samples #1 to #4 increase gradually from 901 to 994 MPa at 650 °C under tensile deformation. The average tensile strength of sample #5 is only 2 MPa greater than that of sample #4. When the fatigue life of the test specimen was tested at 455 °C, the average cycle number of sample #5 was 6 cycles greater than that of sample #4, but 22, 55, and 97 greater than the values for samples #3, #2, and #1, respectively. The tensile strength and fatigue life at room and high temperatures can thus be seen to increase gradually and tend to stabilize as the distance between the sampling region and the edge of the ingot increases. Therefore, it can be tentatively determined that a suitable turning amount for a GH4169 superalloy ingot is 36–48 mm.

The number of inclusions, per unit area, in the alloy largely determines the comprehensive mechanical properties of the material. According to the data, when the material is in service at high temperatures, the volume expansion of the inclusions on or near the surface of the material is caused by oxides, which results in the formation of cracks due to the mismatch between the inclusions and the degree of deformation of the surrounding matrix. This will seriously compromise the mechanical properties and service life of the material [31]. In addition, because of the different properties of the inclusions and matrix, the inclusions hinder the dislocation of the matrix, resulting in dislocation pile-ups in front of the inclusions, as well as local stress concentrations. When the concentrated stress increases with the deformation, ultimately attaining the strength of either the inclusion itself or the interface bonding strength between the inclusion and matrix, the inclusion itself will break or break away from the interface with the matrix, resulting in micro-pore cracking or structural defects, thus limiting the service performance of the alloy [32]. Obviously, to improve the properties of the alloy, it is very important to reduce the number of inclusions per unit area.

## 4. Conclusions

Steel mills rely on operational experience or an ingot’s surface finish as a measure of the turning quality of a VAR ingot, ignoring the slag discharge effect of the VAR process. Even if the surface finish of the ingot satisfies the technological requirements, a large number of types and amounts of inclusions remain on the surface, which seriously limit any further improvement of the alloy performance. Therefore, this study analyzed and compared the types and amounts of inclusions, the average area of the (Al,Mg)O inclusions, and the mechanical properties at different locations relative to the GH4169 superalloy VAR ingot edge. In this way, we systematically studied the effects of the amount of turning to which the superalloy was subjected, the metallurgical qualities, and the mechanical properties.

1. We identified five different types of inclusion in the five groups of samples taken from different regions: Ti(C,N) composite inclusions, Ti(C,N)-Nb composite inclusions, SiC inclusions, Ti(C,N)-(Nb,P,Mo,S) composite inclusions, and (Al,Mg)O-Ti(C,N)-(Nb,P,Mo,S) composite inclusions. With an increase in the distance between the sampling location and the edge of the ingot, the distribution of the number of inclusions first decreases from 1917 to 1310 N/mm^2^. It then fluctuated steadily within a range of 1166 ± 10 N/mm^2^.
2. The average area of the (Al,Mg)O-Ti(C,N)-(Nb,P,Mo,S) composite inclusions decreased from 7.61 to 0.49 μm^2^ and then fluctuated steadily within a range of 0.49 ± 0.02 um^2^ as the distance between the sampling location and the edge of the ingot increased. The evolution process is similar to that of the quantity distribution of the inclusions.
3. As the distance between the sampling location and the edge of the ingot gradually increased each of the five groups, the tensile strength, elongation, section shrinkage at 25 °C, and that at 650 °C, increased from 1102 to 1911 MPa, 901 to 996 MPa, 13.0% to 15.5%, 24.0% to 29.0%, 28.0% to 32.0%, and 55.0% to 60.0%, respectively. At the same time, the fatigue life at 455 °C increased from 7176 to 7273 cycles, and the hardness increased from 442 HB to 448 HB. However, the range of fluctuation of the mean values of the properties of samples #4 and #5 was less than 6.
4. According to the results obtained for the type and quantity distribution of the inclusions in the five groups of samples, the change in the average area of the (Al,Mg)O composite inclusions, and the change trend of the various properties, it can be tentatively determined that a suitable turning amount for a GH4169 superalloy ingot is 36–48 mm.

## Figures and Tables

**Table 1:** Furnace charge proportions of the GH4169 superalloy (wt%).

Ni	Cr	Nb	Ti	Al	Mo	Co	C	Fe
50.0–55.0	17.0–21.0	4.7–5.5	0.6–1.1	0.2–0.8	2.8–3.3	≤1.0	≤0.08	Balance

**Table 2:** Elongation, section shrinkage, and hardness of GH4169 superalloy sample groups.

Mechanical Properties	#1	#2	#3	#4	#5
Elongation (25 °C, %)	13.0 ± 0.5	14.0 ± 0.5	15.0 ± 0.5	15.5 ± 0.5	15.5 ± 0.5
Elongation (650 °C, %)	24.0 ± 0.5	25.5 ± 0.5	28.0 ± 0.5	29.0 ± 0.5	29.0 ± 0.5
Section shrinkage (25 °C, %)	28.0 ± 0.5	30.0 ± 0.5	31.0 ± 0.5	32.0 ± 0.5	32.0 ± 0.5
Section shrinkage (650 °C, %)	55.0 ± 0.5	57.0 ± 0.5	59.0 ± 0.5	60.0 ± 0.5	60.0 ± 0.5
Hardness (HB)	442 ± 1	445 ± 1	446 ± 1	447 ± 1	448 ± 1
